# Effect of intravenous fluid therapy for acute alcohol intoxication on length of time from arrival at the emergency department until awakening: A prospective observational cohort study

**DOI:** 10.1002/ams2.841

**Published:** 2023-05-03

**Authors:** Takero Terayama, Ruka Sasa, Yuka Nakatani, Fumika Tanaka, Sho Terashige, Daishi Higashiyama, Takao Sugiura, Kosuke Hatanaka, Takashi Nishiyama, Shigeto Takeshima

**Affiliations:** ^1^ Department of Emergency Self‐Defense Forces Central Hospital Tokyo Japan; ^2^ Department of Psychiatry, School of Medicine National Defense Medical College Saitama Japan; ^3^ Department of Health Informatics Kyoto University School of Public Health Kyoto Japan

**Keywords:** Acute alcoholic intoxication, ethanol, intravenous fluid therapy, observational study, Ringer's solution

## Abstract

**Aim:**

To evaluate the association of intravenous fluid (IVF) therapy on the length of time from arrival at the emergency department (ED) until awakening in cases of acute alcohol intoxication.

**Methods:**

This single‐center, prospective, observational study was conducted in the ED of the Self‐Defense Forces Central Hospital during October 1, 2018 to July 31, 2019. Patients with 1,000 mL bolus of lactated Ringer's solution and those without bolus were compared. The primary outcome was the length of time until awakening. Secondary outcomes were the length of stay in the ED and occurrence of conditions requiring extra care. Predictors of the occurrence of any event requiring extra care were identified.

**Results:**

We included 201 patients, of whom 109 received IVF and 92 did not. No significant difference existed in the baseline characteristics between the groups. The median length of time until awakening did not significantly differ between the groups (*P* = 0.77). Multivariable regression analysis adjusted by age, sex, hemoglobin, blood alcohol concentration, and initial Glasgow Coma Scale (GCS) score demonstrated that the regression coefficient of IVF for length of time until awakening was −9.55 (95% confidence interval [CI], −36.2 to 17.2). Hemoglobin (regression coefficient, 10.1; 95% CI, 0.38–19.9) and initial GCS score (regression coefficient, −7.51; 95% CI, −10.8 to −4.21) were significantly associated with length of time.

**Conclusion:**

IVF therapy was not associated with the length of time until awakening in patients with acute alcohol intoxication in the ED. Routine IVF administration was unnecessary.

## Graphical abstract

Intravenous fluid therapy (IVF), which is commonly used in patients with acute alcohol intoxication, did not shorten length of time until awakening in the emergency department. Therefore, IVF should not be routinely used for the treatment of these patients.

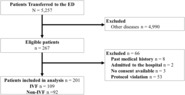

## INTRODUCTION

Acute alcohol intoxication commonly occurs in patients admitted to urban emergency departments (EDs) who present with difficulty walking, vomiting, or loss of consciousness, owing to a rapid increase in blood alcohol concentration (BAC). In Tokyo, ~720,000 cases, including more than 15,000 patients with acute alcohol intoxication, are transported to emergency hospitals annually.^1^ Patients with acute alcohol intoxication are often admitted to the ED for a long time and require more attention from ED staff members, owing to agitation or violent acts; this interferes with the operation of the ED. Therefore, it is essential to provide appropriate treatment approaches for these patients to reduce the length of time until awakening and the frequency of events requiring extra care from ED staff.

In many EDs, intravenous fluid (IVF) therapy is often performed to treat these patients, and IVF is expected to promote blood alcohol clearance and shorten the length of stay in the ED.^2^ Considering mechanisms of alcohol metabolism,^3^, ^4^, ^5^ IVF therapy is not expected to accelerate the rate of alcohol metabolization.^6^ From a clinical standpoint, we hypothesized that routine use of IVF therapy for acute alcohol intoxication would not be effective in the ED. However, evidence of the effectiveness of IVF is limited.^7^, ^8^

Therefore, we conducted a prospective, observational study to evaluate the associations of IVF therapy for acute alcohol intoxication on a new indicator, the length of time from arrival at the ED until awakening.

## MATERIALS AND METHODS

### Study design

This single‐center, prospective, observational study was conducted in the ED of the Self Defense Force Central Hospital (SDFCH), Tokyo, Japan.

### Study setting

Patients were recruited from October 1, 2018 to July 31, 2019. The SDFCH is a 500‐bed, secondary emergency hospital in Tokyo.

### Patient selection

Patients eligible for recruitment into this study were those who were transported to the SDFCH for acute alcohol intoxication, were unable to walk by themselves, and were ages 18 to 69 years. The exclusion criteria were daily use of any psychotropic or sleep medicine, dementia, alcohol addiction, liver cirrhosis, any medical history affecting alcohol metabolism, and lack of consent for participation in this study (Table 1). Patients with head injury, such as brain contusion or intracranial hemorrhage as observed on head computed tomography (CT), were also excluded, as well as patients who were hospitalized for treatment.

**Table 1:** Criteria for patient eligibility

Inclusion criteria	Age, 18–69 years Transported to the hospital in an ambulance Unable to walk
Exclusion criteria	Head injury causing deterioration in consciousness (e.g., brain contusion, intracranial hemorrhage) Daily use of any psychotropic or sleep medicine Dementia Alcohol addiction Liver cirrhosis Any past medical history affecting alcohol metabolism Hospitalized for treatment No consent^a^

^a^ Including cases in which we did not have enough time to explain this study because of crowding in the emergency department.

### Treatment protocol for patients

We predetermined a treatment protocol among ED staff members as follows. The decision to administer IVF treatment was according to the discretion of the attending physician. Subsequently, blood test and blood gas analysis were performed. Patients in the IVF group received a 1,000 mL bolus of lactated Ringer's solution, whereas those in the non‐IVF group underwent only cannulation. Intubation was avoided when impaired consciousness was considered to result from alcohol intoxication.

A head CT scan was performed for patients who could not provide full information from the start of drinking until arrival at the hospital, even if no physical examination of head injury was obtained. When CT was performed, physicians temporarily administered a sedative drug (5 mg diazepam) if necessary, because of agitation.

After all examinations, each patient was monitored for oxygen saturation, blood pressure, and heart rate. Glasgow Coma Scale (GCS) score measurements were performed every 30 min. When patients had no symptoms of agitation and a GCS score of 15 was obtained, nurses examined whether the patient was able to walk without assistance. After a satisfactory walking test result, attending physicians judged whether to discharge the patient and confirmed the walking test and whether the patient would be able to go home alone or escorted by an adult.

When patients left the observation room, the ED staff members explained the study and asked them to participate in this study, and informed consent was obtained. Next, additional short interviews regarding daily consumption of alcohol were conducted. All the attending staff members were trained in this protocol for more than 1 month before the study initiation.

### Outcomes

We measured the length of time until awakening as the primary outcome because it would better reflect the effect of IVF therapy under the robust patient follow‐up protocol in the ED than the length of stay in the ED, which was defined as the primary outcome in prior studies.^7^, ^8^ The length of time until awakening was defined as the period from ED arrival up to the time when the GCS score was 15 and patients were able to walk. We also measured the length of stay in the ED and the occurrence of any event that required extra care by ED staff members, such as vomiting, incontinence, rants, and violence, as secondary outcomes. The length of stay in the ED was defined as the period from ED arrival to the time they left the observation room.

### Variables and measurements

All data were collected from data recording forms of nurses and emergency medical services personnel (Fig. **S1**). These forms included questions about age, sex, vital signs, GCS score at the scene from which patients were transported, initial GCS score in the ED, head CT findings, whether patients were escorted by an adult who was not intoxicated, availability of public transportation from the hospital, outcome (i.e., admission, discharge to home, discharge to police), use of a sedative drug (5 mg of diazepam), occurrence of any event that required extra care from ED staff members, and laboratory test results. The data recording forms, including the amount and type of alcohol, was documented during the interview with the patients and classified into three categories: “seldom,” “sometimes,” and “almost every day.”

### Statistical analysis

Data are presented as means and standard deviations (SDs), numbers and percentages, or medians and interquartile ranges (IQRs). Continuous variables with normal distribution were compared using the Student's *t*‐test. Categorical variables and continuous variables without normal distribution were compared using the χ^2^ test and the Mann–Whitney *U* test, respectively. The association of IVF therapy on the length of stay in the ED was assessed using univariate and multivariate regression analyses, controlled for age, sex, hemoglobin (Hb), BAC, initial GCS score in the ED, daily consumption of alcohol (almost every day), and the presence of escorted adults. β coefficients, and adjusted *R*‐squared values were reported. A multivariate logistic regression model yielding odds ratios (ORs) and 95% confidence intervals (CIs) was used to identify the predictors of the occurrence of any event requiring extra care from ED staff. The model included IVF, age, sex, Hb, BAC, initial GCS score in the ED, and daily consumption of alcohol (almost every day). Subgroup analysis was performed in patients whose daily consumption of alcohol was classified as “almost every day.”

All statistical analyses were performed using SPSS, version 27.0 (IBM Corporation, Armonk, NY). A two‐sided significance level of <0.05 was used, as were 95% CIs. Missing data were not replaced.

## RESULTS

### Participants

**Fig. 1:**
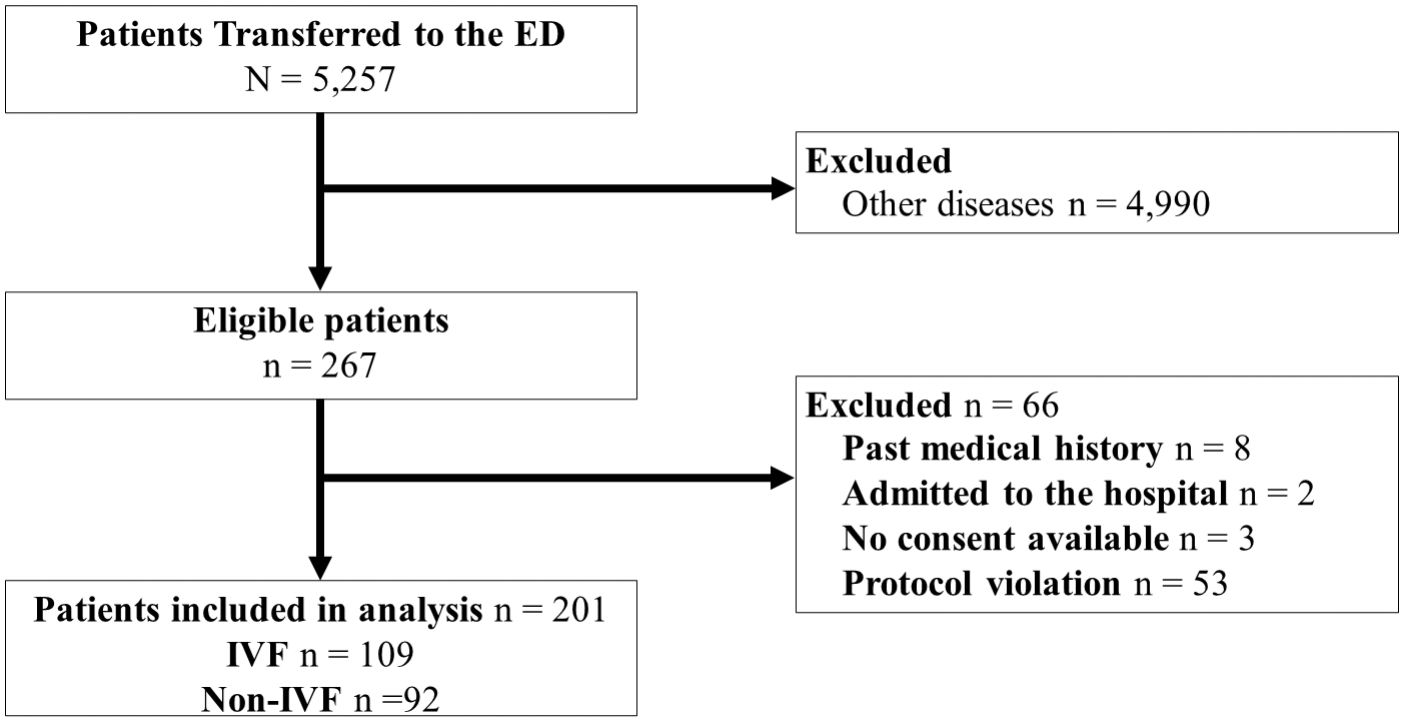
Flowchart of the patient selection process. ED, emergency department; IVF, intravenous fluid.

During the study period, 5,257 patients were transported to the SDFCH. Among those patients, 267 were eligible for inclusion in this study and 201 were included in the final analysis (Fig. 1), and of these, 109 received IVF and 92 did not. There was no significant difference in the baseline characteristics between the groups (Table 2). The median age was 23 (IQR, 21–29) years in the IVF group and 23 (IQR, 21–27) years in the non‐IVF group. All patients in the IVF group received 1,000 mL lactated Ringer's solution, regardless of their body weight. In the subgroup analysis, among patients whose daily consumption of alcohol was classified as “almost every day,” 42 patients were included, of whom 28 received IVF and 14 did not. There was a significant difference in the GCS score at the scene (9 [IQR, 6–12] in the IVF group versus 13.5 [IQR, 8.3–14] in the non‐IVF group; *P* = 0.02), systolic blood pressure (113 mm Hg [IQR, 104–121 mm Hg] versus 101.5 mm Hg [IQR, 96–108.3 mm Hg], respectively; *P* = 0.03), and pH (7.33 [IQR, 7.31–7.35] versus 7.35 [IQR, 7.33–7.44], respectively; *P* = 0.027) (Table S1). There were 66 eligible patients who did not participate, and 53 patients were excluded owing to protocol violation (Fig. 1). Among 53 patients, 24 received IVF therapy. Protocol violation was deemed when the nurses in the ED could not observe patients every 30 min owing to crowding in the ED during the night shift, which also resulted in a few patients not providing consent for participation. A sedative drug was administered to five patients (one in the IVF group and four in the non‐IVF group) once when head CT was performed. For patients included in the final analysis, some variables of laboratory data in a few cases were missing. The subgroup analysis is demonstrated in Table S1. The IVF group had a higher GCS score, systolic blood pressure, and larger proportion of patients who underwent head CT.

**Table 2:** Comparison of baseline characteristics of patients with acute alcohol intoxication between the groups

Characteristics	Non‐IVF	IVF	*P* *
*N*	92	109	
Age (years)	23 (IQR, 21–27)	23 (IQR, 21–29)	0.96
Male sex	60 (65.2%)	68 (62.4%)	0.68
GCS score at the scene^†^	10.5 (IQR, 6–14)	11 (IQR, 6–13)	0.43
No. of patients who arrived at the ED in the middle of night^‡^	47 (51.1%)	70 (64.2%)	0.06
Systolic blood pressure (mm Hg)	105 (IQR, 97–116)	105 (IQR, 98–116)	0.86
Heart rate (per min)	83 (IQR, 76–93)	82 (IQR, 98–116)	0.51
Initial GCS score in the ED	10.5 (IQR, 6–14)	9 (IQR, 6–13)	0.67
Blood gas analysis			
pH	7.33 (IQR, 7.31–7.38)	7.34 (IQR, 7.31–7.37)	0.75
Base excess (mmol/L)	−1.75 (IQR, −3.50 to −0.40)	−2.10 (IQR, −3.50 to −0.70)	0.30
Hb (mg/dL)	14.7 (IQR, 13.5–15.5)	14.4 (IQR, 13.7–15.3)	0.42
BAC (mg/dL)	267 (IQR, 214–322)	246 (IQR, 211–309)	0.15
No. of patients who underwent head CT	26 (28.3%)	31 (28.4%)	0.98
No. of patients who used a sedative drug (5 mg of diazepam)	1 (1.1%)	4 (3.7%)	0.25
No. of patients who were escorted by an adult^§^	55 (61.8%)	74 (69.2%)	0.28
Daily consumption of alcohol			
Seldom	27 (30.7%)	24 (22.9%)	0.16
Sometimes	47 (53.4%)	53 (50.5%)	
Almost every day	14 (15.9%)	28 (26.7%)	
Unknown	4 (4.3%)	4 (3.7%)	

Abbreviations: BAC, blood alcohol concentration; CT, computed tomography; ED, emergency department; GCS, Glasgow Coma Scale; Hb, hemoglobin; IQR, interquartile range; IVF, intravenous fluid.

* χ^2^ or Mann–Whitney U test.

^†^ Measured by emergency service personnel.

^‡^ 22:00–23:59 or 00:00–03:59.

^§^ Responsible and non‐intoxicated adult who could take the patient home.

### Length of time until awakening

The median length of time until awakening was 210 min (IQR, 148–262 min) in the IVF group and 237 min (IQR, 101–328 min) in the non‐IVF group, with an insignificant difference between both groups (*P* = 0.70) (Table 3). Multiple regression analysis showed no association between IVF and length of time (regression coefficient, −9.55; 95% CI, −36.2 to 17.2), whereas baseline Hb (regression coefficient, 10.1; 95% CI, 0.38–19.9) and initial GCS score in the ED (regression coefficient, −7.51; 95% CI, −10.8 to −4.21) were related to length of time (Table 4). The adjusted *R*‐square value for this model was 0.69 and the residuals of the model were normally distributed. In the subgroup analysis, the median length of time until awakening was 210 min (IQR, 148–262 min) in the IVF group and 237 min (IQR, 101–328 min) in the non‐IVF group, with an insignificant difference between both groups (*P* = 0.70) (Table 3).

**Table 3:** Comparison of outcomes between the groups in univariate analysis

Outcomes	Non‐IVF	IVF	*P* *
All patients			
*N*	92	109	
Length of time until awakening^†^ (min)	210 (IQR, 150–300)	210 (IQR, 145–265)	0.77
Length of stay in the ED (min)	245 (IQR, 165–340)	245 (IQR, 188–310)	0.62
Occurrence of any event that required extra care from ED staff members^‡^ (%)	41 (44.6)	50 (45.9)	0.85
Patients who drink alcohol almost every day			
*N*	14	28	
Length of time until awakening^†^ (min)	237 (IQR, 101–328)	210 (IQR, 148–262)	0.70
Length of stay in the ED (min)	266 (IQR, 138–330)	265 (IQR, 180–329)	0.63
Occurrence of any event that required extra care from ED staff members (%)	6 (42.9)	13 (46.4)	0.83

Abbreviations: ED, emergency department; IQR, interquartile range; IVF, intravenous fluid.

* χ^2^ or Mann–Whitney *U* test.

^†^ Defined as the time from ED arrival to achieving Glasgow Coma Scale score of 15 and demonstrating the ability to walk.

^‡^ Vomiting, incontinence, rants, and violence.

**Table 4:** Multiple regression analysis of factors associated with length of time until awakening and length of stay in the ED

Factors	Regression coefficients	95% CI	*P*
Length of time until awakening			
IVF	−9.55	−36.2 to 17.2	0.48
Age (years)	0.33	−1.11 to 1.76	0.65
Male sex	11.2	−17.0 to 39.5	0.43
Hb (mg/dL)	10.1	0.38 to 19.9	0.042
BAC (mg/dL)	−0.18	−0.35 to 0.00	0.052
Initial GCS score in the ED	−7.51	−10.8 to −4.21	0.00
Daily consumption of alcohol (almost every day)	−12.6	−46.4 to 21.3	0.46
Length of stay in the ED			
IVF	−0.31	−32.4 to 31.7	0.98
Age (years)	0.19	−1.53 to 1.90	0.83
Male sex	14.6	−19.5 to 48.7	0.40
Hb (mg/dL)	8.58	−3.23 to 20.4	0.15
BAC (mg/dL)	−0.15	−0.37 to 0.06	0.16
Initial GCS score in the ED	−6.95	−10.9 to −2.97	0.00
Daily consumption of alcohol (almost every day)	4.69	−35.9 to 45.2	0.82
Presence of an escorted adult^†^	9.79	−24.0 to 43.6	0.57

Abbreviations: BAC, blood alcohol concentration; CI, confidence interval; ED, emergency department; GCS, Glasgow Coma Scale; Hb, hemoglobin; IVF, intravenous fluid.

^†^ Responsible and non‐intoxicated adult who could take the patient home.

### Length of stay in the ED

The median length of stay in the ED was 245 min (IQR, 188–310 min) for the IVF group and 245 min (IQR, 165–340 min) for the non‐IVF group, with an insignificant difference between both groups (*P* = 0.62) (Table 3). Multiple regression analysis showed no association between IVF therapy and length of stay in the ED (regression coefficient, −0.31; 95% CI, −32.4 to 31.7), whereas the initial GCS score in the ED (regression coefficient, −6.95; 95% CI, −10.9 to −2.97) was related to the length of stay (Table 4). The adjusted *R* ^2^ value for this model was 0.64 and the residuals of the model were normally distributed. In subgroup analyses, the median length of stay in the ED was 265 min (IQR, 180–329 min) for the IVF group and 266 min (IQR, 138–330 min) for the non‐IVF group, with an insignificant difference between both groups (*P* = 0.63) (Table 3).

### Events that required extra care from ED staff members

These events, especially vomiting, occurred in 44.6% of patients in the IVF group and in 45.9% of patients in the non‐IVF group, with an insignificant difference between both groups (*P* = 0.85) (Table 3). In the logistic regression model for the occurrence of any event, the OR for IVF to non‐IVF was 1.19 (95% CI, 0.66–2.16) after adjustment for predefined covariates (Table 5). In subgroup analysis, the events occurred in 42.9% of patients in the IVF group and 46.4% of patients in the non‐IVF group, with an insignificant difference between both groups (*P* = 0.83) (Table 3).

**Table 5:** Multiple logistic regression analysis of factors associated with the occurrence of any event that required extra care from ED staff members

Factor	Odds ratio	95% CI	*P*
IVF	1.19	0.66–2.16	0.56
Age (years)	1.00	0.97–1.03	0.91
Male sex	1.19	0.63–2.24	0.59
Hb (mg/dL)	1.11	0.89–1.39	0.36
BAC (mg/dL)	1.01	1.00–1.01	0.01
Initial GCS score in the ED	1.01	0.94–1.08	0.86
Daily consumption of alcohol (almost every day)	0.94	0.45–2.00	0.88

Abbreviations: BAC, blood alcohol concentration; CI, confidence interval; ED, emergency department; GCS, Glasgow Coma Scale; Hb, hemoglobin; IVF, intravenous fluid.

## DISCUSSION

This study evaluated the association of IVF therapy for acute alcohol intoxication on the length of time until awakening and found no association. However, Hb and initial GCS score in the ED were significantly associated. These results were in accordance with our hypothesis that IVF therapy would not accelerate alcohol metabolization, based on the mechanism of alcohol metabolism and previous studies.^7^, ^8^ The results suggest that the GCS score is a better indicator reflecting the influence of alcohol on the central nervous system of the individual patient and that IVF therapy does not reduce these effects. The result that higher Hb results in a longer length of time until awakening is consistent with the clinical perspective that dehydration would have a stronger influence of alcohol on the central nervous system, although this concept has not been demonstrated in clinical settings by quantitative measurements.

A previous randomized controlled trial with 144 cases conducted in Australia showed that a bolus of 20 mL/kg of IVF did not shorten the length of stay in the ED,^8^ with the mean length of stay at 287 min (SD, 171 min) for the IVF group and 274 min (SD, 131 min) for the non‐IVF group, with an insignificant difference (*P* = 0.89). However, in that trial, patients ages >50 years or those who experienced trauma were excluded, which would have resulted in less external validity because patients with alcoholic events were likely to experience trauma to some extent. A single‐center, retrospective, cohort study demonstrated that IVF prolonged the length of stay in the ED.^7^ Although the median length of stay in the ED was 254.5 min (IQR, 203–267 min) for the IVF group and 189 min (IQR, 160–230 min) for the non‐IVF group, with an insignificant difference between both groups (*P* = 0.057), Cox proportional hazards regression of the association between the length of time and IVF therapy adjusted for some covariates showed significant differences (hazard ratio, 0.54; 95% CI, 0.35–0.84; *P* = 0.006). However, this study did not account for some important factors that may affect the association of IVF, such as total infusion volume, infusion speed, dehydration, and observation protocol. These would largely confound the results.

This study had some strengths. First, the primary outcome was the length of time until awakening, which would not be affected by various factors unlike the length of stay in the ED. Second, we devised a treatment protocol in advance, in which attending nurses performed repeated measurements of the GCS score at shorter intervals, which increased the accuracy of the evaluation of the IVF effect. The length of stay in the ED in this study was shorter than that in the previous two studies.^7^, ^8^ Third, patients with minor trauma were also included, and BAC was measured in all cases, which would have better external validity than that in the previous studies.^7^, ^8^ Last, patients’ alcohol‐metabolizing enzymes, which would play a key role of metabolizing alcohol, was not evaluated.

### Limitations

This study had several limitations. First, the insufficiency of infusion dose may reduce the effect of IVF. Because of the location of our hospital, various patients, including those from different nationalities, were transported. Therefore, it was difficult to obtain enough information about medical history and, in view of safety, the dose of IVF was set at 1,000 mL. Second, although we considered the capacity of metabolizing alcohol using daily consumption of alcohol, the daily consumption of alcohol would be an inadequate index for the capacity of metabolizing alcohol. We did not consider the period from intake of alcohol until the measurement of BAC, as well as both amount and type of alcohol. These were important factors to investigate the effect of IVF, but were difficult to obtain in the ED because patients often did not remember them. Third, this study was limited by its single‐center design and small sample size.

## CONCLUSIONS

Intravenous fluid did not influence the length of time until awakening in patients with acute alcohol intoxication. Our results demonstrated that routine use of IVF therapy for acute alcohol intoxication should be avoided.

## DISCLOSURE

Approval of the Research Protocol: ethics approval was obtained from the Ethics Committee of SDFCH on October 1, 2018 (license ID 30‐010).

Informed Consent: Written informed consent was obtained from all patients.

Registry and the Registration No. of the study/trial: Not applicable.

Animal Studies: Not applicable.

Conflict of Interest: None.

## Supporting information


**Table S1.** Comparison of baseline characteristics of patients with acute alcohol intoxication who drink alcohol almost every day.
**Figure S1.** Record form of nurses for this study.
