# Supplementary material for: Effect of intravenous fluid therapy for acute alcohol intoxication on length of time from arrival at the emergency department until awakening: A prospective observational cohort study
Source: Acute Med Surg. 2023 May 3;10(1):e841. doi: 10.1002/ams2.841 (PMC10156605; doi:10.1002/ams2.841)
Supplement: Supplementary file 1 — Table S1. Comparison of baseline characteristics of patients with acute alcohol intoxication who drink alcohol almost every day. Figure S1. Record form of nurses for this study. [file AMS2-10-e841-s001.docx]

**SUPPORTING INFORMATION**

**Table S1.** Comparison of baseline characteristics of patients with acute alcohol intoxication who drink alcohol almost every day

| **Characteristics** | **Non-IVF** | **IVF** | ***p*^*^** |
| --- | --- | --- | --- |
| *N* | 14 | 28 |  |
| Age (years) | 29 (IQR, 25-53) | 25 (IQR, 22-42) | 0.23 |
| Male sex | 10 (71.4%) | 21 (75.0%) | 0.80 |
| GCS score at the scene^†^ ^k^ | 13.5 (IQR, 8.3‒14) | 9 (IQR, 6‒12) | 0.02 |
| No. of patients who arrived at the ED in the middle of night^‡^ | 8 (57.1%) | 20 (71.4%) | 0.36 |
| Systolic blood pressure (mmHg) | 101.5 (IQR, 96‒108.3) | 113 (IQR, 104‒121) | 0.03 |
| Heart rate (per min) | 81.5 (IQR, 75‒87.8) | 80 (IQR, 76‒87) | 0.68 |
| Initial GCS score in the ED | 11 (IQR, 6‒14) | 10 (IQR, 7‒15) | 0.55 |
| Blood gas analysis |  |  |  |
| pH | 7.35 (IQR, 7.33‒7.44) | 7.33 (IQR, 7.31‒7.35) | 0.03 |
| Base excess (mmol/L) | -3.0 (IQR, -3.85‒-0.403.3) | -2.1 (IQR, -3.2‒-1.2) | 0.86 |
| Hb (mg/dL) | 15.1 (IQR, 13.4‒15.4) | 14.4 (IQR, 13.9‒15.2) | 0.74 |
| BAC (mg/dL) | 258 (IQR, 199‒296) | 288 (IQR, 214‒324) | 0.34 |
| No. of patients who underwent head CT | 1 (7.1%) | 9 (32.1%) | 0.07 |
| No. of patients who used a sedative drug | 0 (0.0%) | 2 (7.1%) | 0.31 |
| No. of patients who were escorted by an adult^§^ | 7 (50.0%) | 17 (63.0%) | 0.42 |

IVF, intravenous fluid; IQR, interquartile range; GCS, Glasgow Coma Scale; ED, emergency department; Hb, hemoglobin; BAC, blood alcohol concentration; CT, computed tomography

^*^Chi-square or Mann–Whitney U test

^†^Measured by emergency service personnel

^‡^22:00–23:59 or 00:00–03:59

^§^Responsible and non-intoxicated adult who could take the patient home

**Figure S1.** Record form of nurses for this study.


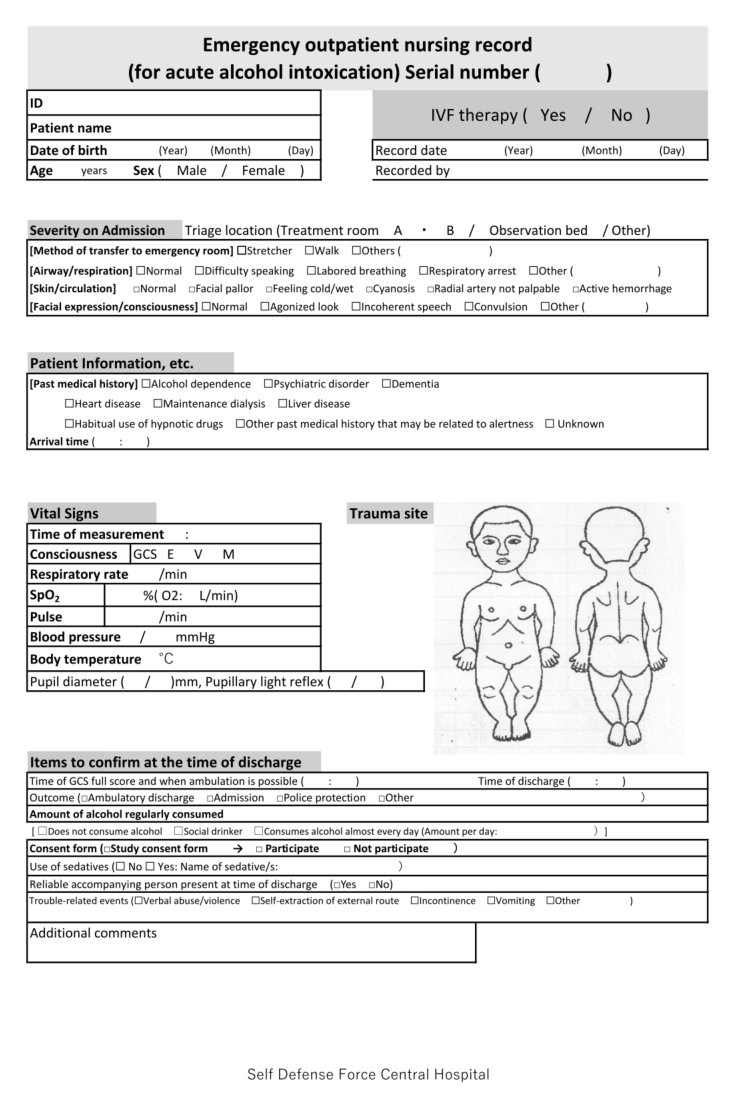


IVF, intravenous fluid; GCS, Glasgow Coma Scale
